# Next Generation Sequencing Reveals the Expression of a Unique miRNA Profile in Response to a Gram-Positive Bacterial Infection

**DOI:** 10.1371/journal.pone.0057543

**Published:** 2013-03-05

**Authors:** Nathan Lawless, Amir B. K. Foroushani, Matthew S. McCabe, Cliona O’Farrelly, David J. Lynn

**Affiliations:** 1 Animal and Bioscience Research Department, Animal and Grassland Research and Innovation Centre, Teagasc, Grange, Dunsany, County Meath, Ireland; 2 School of Biochemistry and Immunology, Trinity College, Dublin, Ireland; 3 Department of Molecular Biology and Biochemistry, Simon Fraser University, Burnaby, British Columbia, Canada; University of California, Davis, United States of America

## Abstract

MicroRNAs (miRNAs) are short, non-coding RNAs, which post-transcriptionally regulate gene expression and are proposed to play a key role in the regulation of innate and adaptive immunity. Here, we report a next generation sequencing (NGS) approach profiling the expression of miRNAs in primary bovine mammary epithelial cells (BMEs) at 1, 2, 4 and 6 hours post-infection with *Streptococcus uberis*, a causative agent of bovine mastitis. Analysing over 450 million sequencing reads, we found that 20% of the approximately 1,300 currently known bovine miRNAs are expressed in unchallenged BMEs. We also identified the expression of more than 20 potentially novel bovine miRNAs. There is, however, a significant dynamic range in the expression of known miRNAs. The top 10 highly expressed miRNAs account for >80% of all aligned reads, with the remaining miRNAs showing much lower expression. Twenty-one miRNAs were identified as significantly differentially expressed post-infection with *S. uberis*. Several of these miRNAs have characterised roles in the immune systems of other species. This miRNA response to the Gram-positive *S. uberis* is markedly different, however, to lipopolysaccharide (LPS) induced miRNA expression. Of 145 miRNAs identified in the literature as being LPS responsive, only 9 were also differentially expressed in response to *S. uberis*. Computational analysis has also revealed that the predicted target genes of miRNAs, which are down-regulated in BMEs following *S. uberis* infection, are statistically enriched for roles in innate immunity. This suggests that miRNAs, which potentially act as central regulators of gene expression responses to a Gram-positive bacterial infection, may significantly regulate the sentinel capacity of mammary epithelial cells to mobilise the innate immune system.

## Introduction

MicroRNAs (miRNAs) are an abundant class of highly conserved, small (19–24 nt long), non-coding, double-stranded RNA molecules. They act as post-transcriptional regulators of gene expression, altering mRNA stability and translation efficiency by hybridizing to the 3′ untranslated regions (UTRs) of certain subsets of mRNAs (collectively as many as 60% of all mRNA transcripts) [1]. Since their initial discovery in *Caenorhabditis elegans* in 1993 [2], researchers have gained much insight into the prevalence of miRNAs in other species. The latest miRBase database (release 19) contains 21,264 precursor miRNAs, expressing 25,141 mature miRNA products, in 193 species [3].

miRNAs have been shown to play key roles in the regulation of innate and adaptive immunity in humans and mice [4]. miR-146a, for example, regulates the innate immune response to bacterial infection, targeting TNF receptor-associated factor 6 (TRAF6) and Interleukin-1 receptor-associated kinase 1 (IRAK1) [5], while miR-150 regulates the production of mature B cells [6]. Studies elucidating the regulatory roles of miRNAs in bovine infection and immunity, however, are more limited. Bovine miRNAs have been shown to be expressed in a wide range of tissues, including immune-related ones [7], , but only a handful of studies have investigated how the expression of bovine miRNAs are altered in response to infection. A recent RT-qPCR study, for example, highlighted the differential expression of five inflammation related miRNAs (miR-9, miR-125b, miR-155, miR-146a and miR-223) in response to *E. coli* lipopolysaccharide (LPS) and *S. aureus* enterotoxin B stimulation of bovine monocytes [10]. Two other recent studies have used a similar approach to identify several miRNAs that were differentially expressed in the mammary gland tissue of cattle with mastitis [11], [12]. These and other studies suggest roles for individual miRNAs in regulating bovine immunity, however, according to Ensembl v66 [13], [14] there are over 1,300 annotated miRNAs in the bovine genome. Therefore, studies which adopt genome-wide approaches are required to gain greater insight into the repertoire of bovine miRNAs involved in immunity and infection.

Although microarray technologies to profile miRNA expression have been around for some time [15], next generation sequencing (NGS) based technologies are revolutionising the field and provide the opportunity to profile the expression of known miRNAs with discriminating resolution and accuracy, and also to identify novel miRNAs [16]. Furthermore, these technologies allow one to differentiate between the expression of alternative mature miRNAs from the same precursor and to identify the differential expression of miRNA isomiRs [17]. To date, a limited number of studies have applied these approaches to profile miRNAs in different bovine tissues [18], [19], [20] and only one study has used an NGS approach to investigate the expression of bovine miRNAs in response to infection [21].

In this study, we implemented a NGS approach to profile the expression of bovine miRNAs at multiple time-points in primary mammary epithelial cells infected *in vitro* with *Streptococcus uberis*, a causative agent of bovine mastitis. This inflammatory disease of the mammary gland has significant economic impact on the global dairy industry [22]–[23]. To the best of our knowledge, this study represents the most comprehensive NGS study to date that profiles the host miRNA response to infection, in any species. In comparison to previous studies, we have sequenced un-pooled miRNA libraries to a previously unprecedented sequencing depth from multiple replicates and controls across multiple time-points, allowing us to explore the statistically significant temporal changes in miRNA expression in response to infection.

## Materials and Methods

### Bovine Mammary Epithelial Cell Culture

Primary bovine mammary epithelial cells, which had been isolated from mammary parenchyma, were purchased from AvantiCell (AvantiCell Science Ltd., Ayr, UK) [24], [25]. The source animal was in her third trimester of first pregnancy, was aged between 26–30 months, and was negative for bovine viral diarrhoea and Bovine spongiform encephalopathy. Cells were plated (seed density of 1×10^6^) directly onto collagen coated plastic flasks (Greiner-Bio-One GmbH, Frickenhausen, Germany) and immersed in AvantiCell medium – (199/Ham’s F12 (50∶50) pH 7.4 containing 5% (v/v) horse serum, 5% (v/v) fetal bovine serum, 5 µg/ml bovine insulin, 1 µg/ml hydrocortisone, 3 µg/ml cortisol, 10 ng/ml epidermal growth factor (EGF), 2 mM sodium acetate, 10 mM Hepes, U/ml penicillin/streptomycin and single strength Fungizone™).

Media was initially replaced after 48 h. Cells were split twice (75 cm^2^ and 175 cm^2^) and were then seeded at a concentration of 1.8×10^5^ cells/well into collagen coated 6-well plates. Media was then changed after 24 h, and cells were inspected under microscopy for confluence. Cells were harvested by washing with Hanks Balanced Salt Solution (HBSS) pH 7.4 and treated with 4 ml of 0.25% trypsin for approximately 5 min at 37°C. An equal volume of medium to trypsin (1∶1) neutralised trypsin.

### Infection of Cells with *Streptococcus uberis* 0140J

*Streptococcus uberis* 0140J was purchased from the American Type Culture Collection (ATCC), Virginia, USA (Cat# BAA-854). *S. uberis* 0140J was first isolated in milk obtained from a clinical case of bovine mastitis in the United Kingdom in 1972. *S. uberis* was cultured as per ATCC instructions. BMEs were challenged with *S. uberis* 0140J at a multiplicity of infection (MOI) of 50, over a time course of 1, 2, 4, & 6 h. Three replicates were infected at each time point and three replicate uninfected controls were also maintained for each time point.

### miRNA Extraction

Total RNA and small RNA was extracted from each of the 24 samples using the mirVana™ miRNA Isolation Kit (Life Technologies, Carlsbad, CA, USA). Procedures were performed according to the manufacturer’s protocol. Briefly, cells were lysed using 500 µl lysis/binding solution directly on a culture plate. 50 µl of miRNA homogenate was added; solution was mixed by vortexing and left on ice for 10 min. 500 µl of acid-phenol chloroform was added and solution was mixed by vortexing for ∼60 sec. The solution was then centrifuged for 5 min at 10,000×g at room temperature to separate phases. Aqueous phase was removed and transferred to a separate tube. 1/3 volume of 100% ethanol was added to the aqueous phase and mixed by vortexing. Samples were passed through a filter cartridge (glass-fiber filter) by centrifuge for ∼30 sec at 10,000×g. The filtrate was collected (residue on filter contained RNA <200 nt, was retained for later use) and 2/3 volume 100% ethanol was added and mixed by vortexing. Filtrate was passed through a second filter cartridge by centrifuge for ∼30 sec at 10,000×g. The flow through was discarded, and the filter was washed with 700 µl wash solution 1 and 500 µl wash solution 2 (twice). After discarding all flow through after each step, the filter was centrifuged for a further 1 min. 50 µl of pre-heated (95°C) nuclease free water was applied to the filter for 1 min, and the filter was centrifuged for 30 sec. Eluate was collected and stored at −80°C. Total RNA integrity was measured by the Agilent RNA 6000 Nano Kit using the 2100 Bioanalyzer (Agilent Technologies, Colorado Springs, CO, USA). The Agilent Small RNA Kit (Agilent Technologies) was used to quantify miRNA.

### Small RNAseq Library Preparation and Sequencing

Twenty-four indexed miRNA libraries were prepared using the ScriptMiner™ Small RNAseq Library Preparation Kit (Epicentre, Madison, WI, USA). Procedures were performed according to the manufacturer’s protocol. Briefly, a 3′-tagging sequence was added to the 3′- end of the RNA followed by treatment with a degradase enzyme to reduce excess 3′ adaptor oligo. A tagging sequence was then added to the 5′- end of the RNA. The RNA, now tagged at both ends (di-tagged), was purified using the Zymogen RNA Clean and Concentrator (Zymogen, Irvine, CA, USA). The di-tagged RNA was then reverse transcribed into cDNA, and the remaining RNA was removed using RNase. The PCR step used by the ScriptMiner™ kit, is a two stage process, firstly an analytical PCR step was carried out to optimise the number of cycles necessary for amplification. Once this was determined, the libraries were amplified and adaptors were added. Size fractionation of the miRNA libraries to separate them from adapter dimers was achieved by electrophoresis on an 8% TBE polyacrylamide gel (Life Technologies, Carlsbad, CA, USA) (1.00 mm×10 well). The libraries were then purified from the gel and the Agilent High Sensitivity DNA Kit (Agilent Technologies, Colorado Springs, CO, USA) was used to quantify the molarity and size of finished miRNA-seq libraries. miRNA libraries were randomised across three lanes of a flowcell, with eight indexed samples on each lane. Libraries were sequenced on an Illumina HiSeq 2000 by the Norwegian Sequencing Centre with TruSeq v3 reagents. Fastq files were produced using the CASAVA pipeline v1.8.2. Barcodes (indexes) and adaptor sequences for multiplexed samples are provided (Table S1).

### Small RNAseq Analysis

Preliminary quality control analysis of the 24 fastq files was carried out with FASTQC software v0.10.0 (http://www.bioinformatics.babraham.ac.uk/projects/fastqc/). Cutadapt v1.1 (http://www.cutadapt/) was then used to trim 3′ adaptor sequences. Reads which were shorter than 18 nucleotides after trimming were discarded. Trimmed reads were then further filtered using the fastq quality filter (http://hannonlab.cshl.edu/fastx_toolkit/) v0.0.13. Reads where at least 50% of the bases had a Phred score <20 were removed [26]. Finally, reads passing all the above filters were also trimmed at their ends to remove low quality bases (Phred score <20). Reads which successfully passed filtering were aligned to the bovine genome (UMD3.1) using novoalign version 2.07.11 (http://www.novocraft.com) using the “-m” miRNA mode. Reads that did not uniquely align to the genome were discarded. HTSeq version 0.5.3p3 (http://www-huber.embl.de/users/anders/HTSeq/doc/overview.html) using the union model was used to assign uniquely aligned reads to Ensembl (v66) bovine gene and miRNA annotation (separately).

miRNAseq fastq files have been submitted to the NCBI Gene Expression Omnibus (GEO) database [27] with experiment series accession number GSE41278.

### Differential Expression Analysis

Prior to assessing differential expression, count data were first normalised across libraries using either the trimmed mean of M-values (TMM) normalisation method [28] or upper-quantile normalisation [29]. Differential expression analysis of miRNAseq data has been shown to be sensitive to the normalisation approach implemented [30]. To address this issue, we identified differentially expressed miRNAs in three alternatively normalised datasets; TMM-normalised, upper-quantile normalised and no normalisation. Only miRNAs which were identified as differentially expressed across all three datasets were considered further i.e. the differential expression of these miRNAs was robust to the normalisation procedure. As an aside, we found that the two different normalisation approaches resulted in very similar miRNAs being detected as differentially expressed.

The R (version 2.14.1) Bioconductor package EdgeR (v2.4.6) [28], which uses a negative binomial distribution model to account for both biological and technical variability was applied to identify statistically significant differentially expressed miRNAs. Only miRNAs that had at least 1 count per million in at least 3 samples were analysed for evidence of differential gene expression. The analysis was undertaken using moderated tagwise dispersions. Differentially expressed miRNAs were defined as having a Benjamini and Hochberg [31] corrected P value of <0.05.

### Novel miRNA Discovery

In addition to profiling the expression of known miRNAs, miRNAseq data can also be used to identify the expression of potentially novel miRNAs. To do this, miRNAseq data from this study was analysed using the software package miRDeep2 v0.0.5 [32]. The miRDeep2 algorithm mines high-throughput sequencing data for the presence of multiple sequenced RNAs corresponding to predicted miRNA hairpin structures in the genome. It then uses Bayesian statistics to score the fit of sequenced RNAs to the biological model of miRNA biogenesis. MiRDeep2 predicted a large number of potentially novel miRNAs from our miRNAseq data. We further parsed this data using a number of different parameters to identify those novel miRNAs that have the highest likelihood of being true positives. Specifically, we identified those predictions where both the mature and star stands were expressed with a minimum of 5 reads each; where miRDeep2 predicted that the miRNA had >90% probability of being a true positive; where the hairpin structure had a significant Randfold p-value and where the novel miRNA was independently predicted in two or more different miRNAseq samples.

Customised Perl scripts were also written in house to examine the miRDeep2 output for the presence of miRNA isomiRs. These scripts were used to identify isomiRs that were expressed at a level of at least 100 reads and to identify cases where the expression of the isomiR was higher than the expression of the miRBase consensus mature sequence. Furthermore, we identified whether isomiRs were modified at the 5′ or 3′ ends (first and last 5 nucleotides). IsomiRs with >1 mismatch to the reference sequence were excluded from the analysis.

### miRNA Target Predictions

Target genes that are potentially regulated by differentially expressed miRNAs were predicted using the consensus of two computational approaches, miRanda v3.3a [33] and TargetScan v6.2 [34], [35], [36]. Given the high false positive rates for miRNA target prediction, we identified only those potential target genes that were predicted by both methods. More specifically, we first established a broad pool of potential targets by applying miRanda to bovine mature miRNA (miRBase v18) and cDNA sequences (UMD3.1, Ensembl v66) under default threshold settings. This resulted in the prediction of thousands of possible target genes per differentially expressed miRNA. To narrow down this pool of potential targets, we used TargetScan to independently identify conserved targets with a PCT-score above 0.9 and/or non-conserved targets with a context+ score above 90%. Target genes that were not corroborated by one of the two methods were discarded. Pathway analysis of predicted gene targets was undertaken using the SIGORA R package (http://cran.r-project.org/web/packages/sigora/index.html) with KEGG pathway annotations [37]. Genes that have an annotated role in innate immunity were identified using www.innatedb.com [38], a curated database of innate immunity genes, pathways and molecular interactions.

## Results

### Isolation of Small RNA from Bovine Mammary Epithelial Cells

Small RNA was isolated for NGS sequencing from *S. uberis* infected primary bovine mammary epithelial cells at 1, 2, 4 and 6 hours post-infection (hpi) (n = 3 infected and n = 3 controls at each time-point). Total RNA and small RNA were examined for quantity and integrity in each of the 24 samples (Table S2). Total RNA was assessed to be of high quality based on both Bioanalyzer and 28S/18S analysis. RNA integrity numbers (RIN) for total RNA were >8 for each sample. The concentration of miRNA in each sample was also assessed (Table S2). Sufficient quantities were present to proceed with RNAseq library preparation.

### High-throughput Sequencing of Small RNA Libraries Prepared from Bovine Mammary Epithelial Cells

Small RNA libraries were prepared from size selected RNA (<200 nucleotides). Libraries were prepared using the ScriptMiner™ protocol with indexing before cluster generation, sequencing and imaging on an Illumina Hiseq 2000. Samples were randomly multiplexed over 3 flowcell lanes for sequencing. Sequencing of small RNA libraries yielded more than 450 million raw sequence reads from mammary epithelial cells. Following a pipeline of adaptor removal, quality filtering and the removal of sequences that were too short, more than 213 million reads were retained for further analysis (78,604,161 and 134,850,887 for control and infected replicates, respectively). These filtered reads were then aligned to the reference *Bos taurus* UMD 3.1 genome. Over 116 million reads aligned uniquely to the genome (Table S1). Reads that aligned to more than one position in the genome were discarded. Uniquely aligning reads were then assigned to known miRNAs using HTseq (http://www-huber.embl.de/users/anders/HTSeq/doc/overview.html) based on Ensembl v66 [13], [14] annotation of the bovine genome.

### Repertoire of RNA Species in Small RNA Libraries

**Figure 1:**
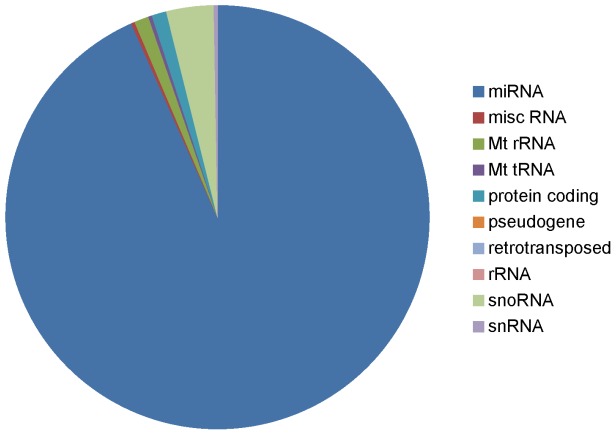
The proportion of reads aligning uniquely to bovine ncRNAs (averaged across 24 samples). The vast majority of reads aligned to known miRNAs.

The proportion of reads (averaged across 24 samples) uniquely aligning to different RNA biotypes demonstrates that miRNAs are the dominant ncRNA species sequenced in our small RNA libraries (Figure 1). The vast majority (>90%) of reads that align uniquely to known ncRNAs align to known miRNAs. There was no significant difference in the proportion of reads aligning to different RNA biotypes in the infected and control samples. The majority of the remaining reads primarily mapped to snoRNAs (Figure 1) [20]. Although the vast majority of reads align to known bovine ncRNAs, a low density of reads can be observed along each chromosome (Figure S1). These possibly represent mRNA degradation products.

### The Expression of miRNAs in Primary Bovine Mammary Epithelial Cells

**Figure 2:**
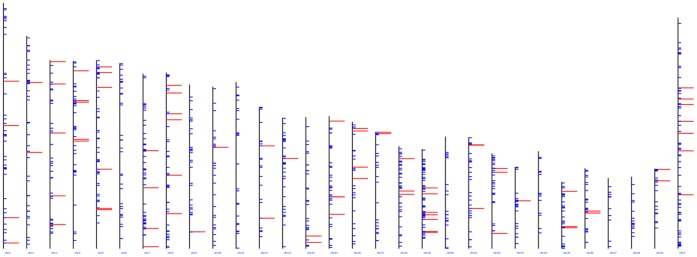
The genomic position of bovine mammary epithelial cell expressed miRNAs with >100 tpm (red). The position of other annotated bovine miRNAs is shown (blue).

To characterise the bovine mammary epithelial cell microRNome, miRNAs that were expressed at an appreciable level (based on mapped read counts in tags per million sequenced (tpm)) were identified. 276 miRNAs had a count of greater than 1 tpm (Table S3). Of these, 114 miRNAs were expressed at a level >100 tpm. To determine whether these miRNAs were expressed from related genomic regions, we examined all miRNAs with >100 tpm for genomic clustering (Figure 2). There was no evidence of a substantial genomic bias from which these miRNAs were encoded.

**Figure 3:**
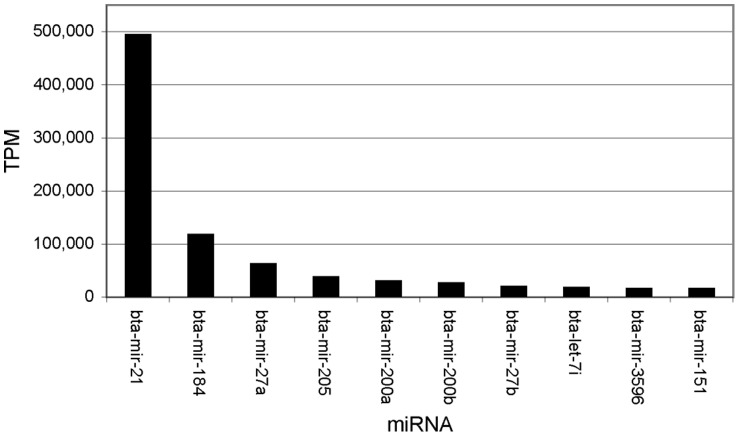
The top 10 most highly expressed miRNAs is bovine mammary epithelial cells.

The top 10 highly expressed miRNAs, which accounted for >80% of all aligned reads (Figure 3), were evolutionarily conserved across multiple species. These miRNAs represent seven different miRNA families; miR-let-7 (bta-let-7i & bta-miR-3596), miR-21 (bta-miR-21), miR-27 (bta-miR-27a & bta-miR-27b), miR-28 (bta-miR-151), miR-184 (bta-miR-184), miR-200 (bta-miR-200a & bta-miR-200b), and miR-205 (bta-miR-205). Many of these miRNAs have been shown to have pleiotropic roles in other species (Table 1). miR-21 and miR-205, have been shown to have role in cancer, regulating tumour suppressor genes such as VEGF-A and TGFI-R2 [39], [40], [41], [42].

**Table 1:** Highly expressed miRNAs in bovine mammary epithelial cells have been shown to have pleiotropic functions in other species.

miRNA	Species	Tissue	Target	Function	Reference
miR-21	Human	Monocytes	CAMP/DEFB4A	Immune	[45]
miR-21	Human	Colon Cancer Cell	TGFI-R2	Cancer	[42]
miR-184	Human	HeLa/HEK	SHIP2	Immune	[46]
miR-205	Human	MCF-7, MDA-MB-231, MDA-MB-453 and MDA-MB-468 cells	VEGF-A	Cancer	[39]–[40]
miR-27b	Human	Monocytes	PPARgamma	Immune	[43]

Of particular interest to our study is the fact that several of the most highly expressed miRNAs in BMEs have been shown to have a role in immunity. miR-27b, for example, has been shown to negatively regulate the mRNA stability of peroxisome proliferator-activated receptor gamma (PPARgamma), a transcriptional regulator of the inflammatory response [43]. Interestingly, miR-27b has also been found to be degraded by a viral transcript in lytic murine cytomegalovirus (MCMV) infection, further highlighting its role in immunity [44]. miR-21 has also recently been shown to be the most highly expressed miRNA in *Mycobacterium leprae* infected monocytes and to negatively regulate the Vitamin D-dependent antimicrobial pathway [45]. Evidence from previous studies also suggests that highly expressed miRNAs in BMEs may also regulate each other. miR-184, for example, has been demonstrated to antagonise miR-205 to maintain SHIP2 levels in epithelia [46].

### Multiple miRNAs are Differentially Expressed in Response to *S. uberis* Infection

Once we had characterised which miRNAs were expressed in unchallenged bovine mammary epithelial cells, we then utilised the EdgeR statistical package [28] to determine which miRNAs were significantly differentially expressed in response to *S. uberis* infection at 1, 2, 4 and 6 hpi. It has been suggested that differential expression analysis of miRNAseq is sensitive to the normalisation approach implemented [30]. To address this issue, we identified differentially expressed miRNAs in three alternatively normalised datasets; TMM-normalised [29], upper-quantile normalised and no normalisation. Only miRNAs which were identified as differentially expressed across all three datasets were considered as significantly differentially expressed i.e. the differential expression of these miRNAs was robust to the normalisation procedure. We found that the two different normalisation approaches actually resulted in very similar miRNAs being detected as differentially expressed.

**Figure 4:**
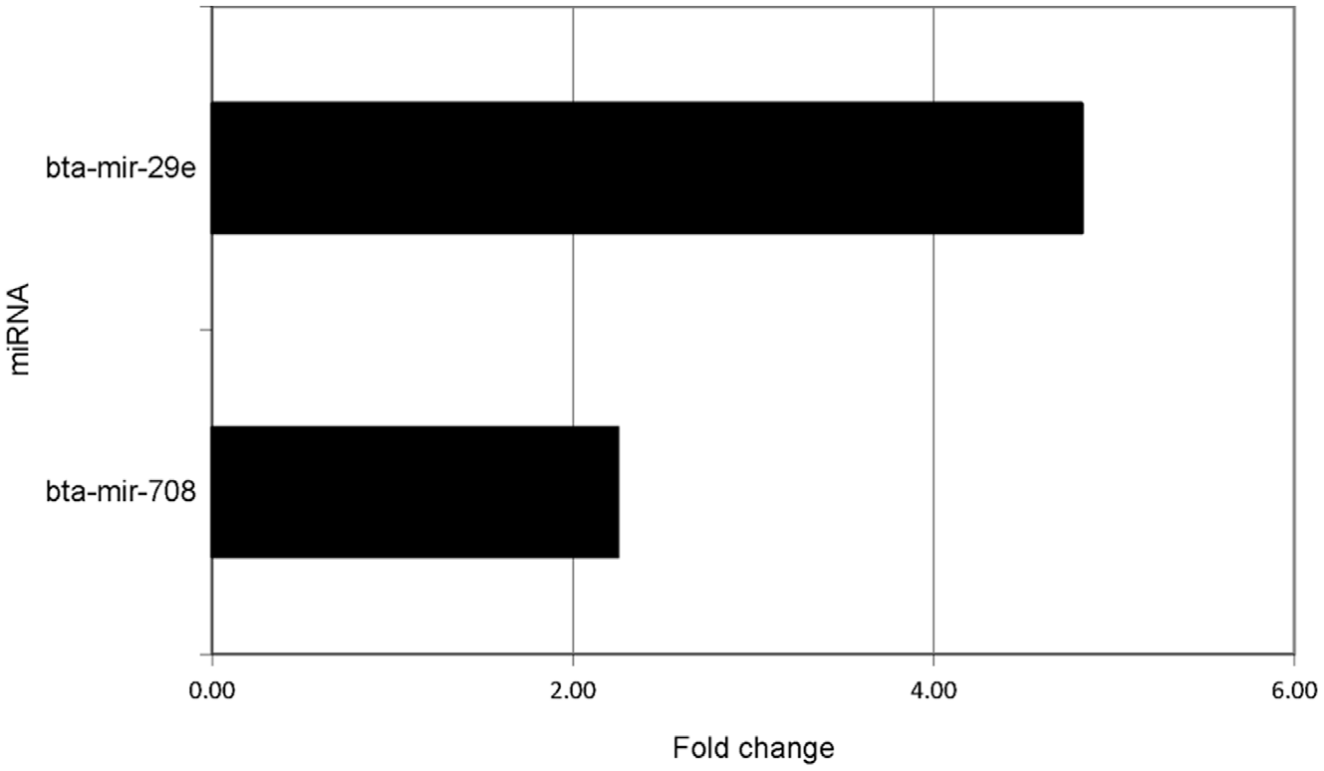
Differentially expressed miRNAs at 2 hours post-infection (hpi).

**Figure 5:**
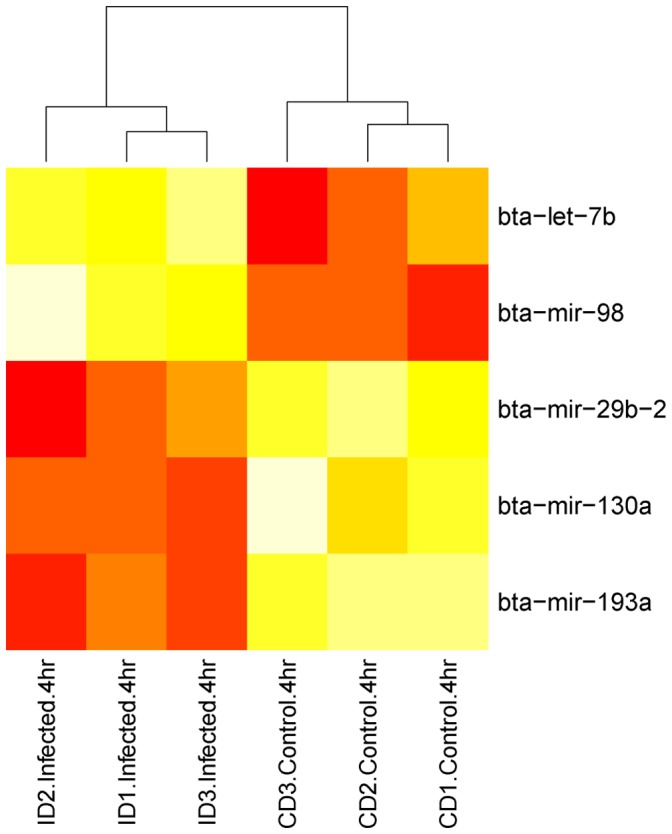
Heatmap of miRNA expression (tpm) across infected and control replicates for each 4 hpi differentially expressed miRNA. The more red the color the more highly expressed that miRNA is. The heatmap was generated using the R v2.14.1 heatmap package.

**Figure 6:**
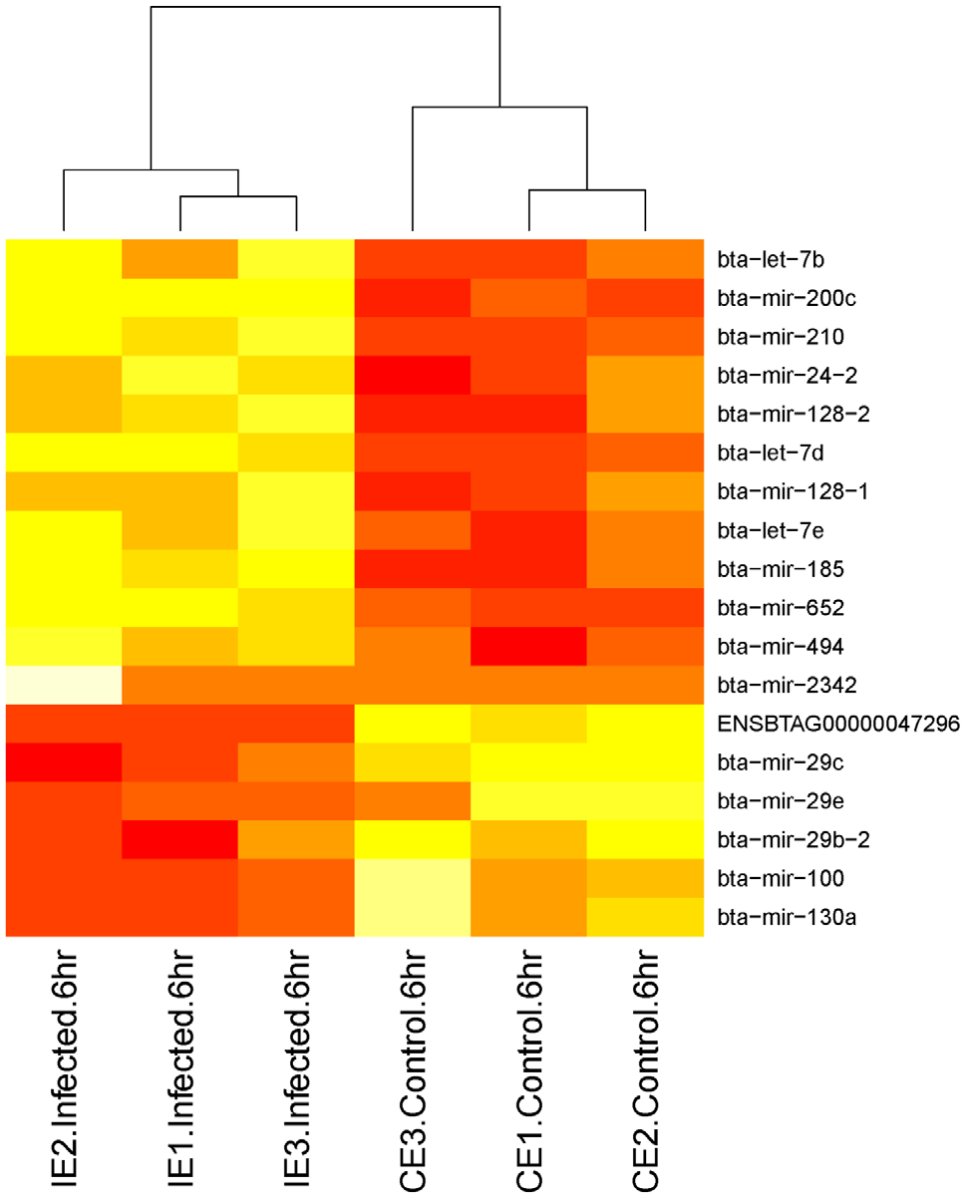
Heatmap of miRNA expression (tpm) across infected and control replicates for each 6 hpi differentially expressed miRNA. The more red the color the more highly expressed that miRNA is. The heatmap was generated using the R v2.14.1 heatmap package.

Fifteen different miRNAs were identified as being significantly up-regulated in response to the *S. uberis* challenge. No miRNAs were identified as differentially expressed at 1 hpi. At 2 hpi, 2 miRNAs, bta-mir-29e and bta-mir-708, were found to be up-regulated (Figure 4). bta-mir-29e was subsequently observed to be down-regulated at 6hpi. At 4 hpi, bta-let-7b and bta-miR-98 were up-regulated (Figure 5). Additionally, bta-miR-let-7c and bta-miR-708 were observed to be up-regulated at 4 hpi when the miRNAseq count data were normalised (both methods), but this was not observed in the un-normalised data. At 6 hpi, 12 miRNAs were found to be up-regulated (Figure 6), including bta-let-7b, which was also up-regulated at 4 hpi.

Seven different miRNAs were identified as down-regulated in response to the *S. uberis* challenge. No miRNAs were down-regulated at 1 or 2 hpi. At 4 hpi, bta-miR-29b-2, bta-miR-193a, and bta-miR-130a were down-regulated. At 6 hpi, bta-miR-29b-2, bta-miR-29c, bta-miR-29e, bta-miR-100, bta-miR-130a and Ensembl predicted miRNA ENSBTAG00000047296, were down-regulated. Two miRNAs, bta-miR-29b-2 and bta-miR-130a were down-regulated at both 4 and 6 hpi. Additionally, bta-miR-15a, bta-miR-17, bta-miR-26a-2, bta-miR-29a, bta-miR-29b-1, and bta-miR-193a were identified as down-regulated in the normalised data (both methods), but not in the un-normalised data. Fold changes in expression for miRNAs that are differentially expressed at 4 and 6 hpi are shown in Figure S2 and S3.

These results indicate that there are rapid temporal changes in the expression of miRNAs in response to a Gram-positive infection, with different miRNA repertoires being identified as differentially expressed at time-points that are 2 hours apart.

### The miRNA Response to the Gram-positive *S. uberis* is Markedly Different to the LPS miRNA Response

To date, many immune-relevant miRNAs have been identified as part of the host response to lipopolysaccharide (LPS) stimulation [47], [48], which is frequently used to mimic a Gram-negative bacterial infection. We have completed a literature survey and identified over 145 miRNAs that have been shown to be differentially expressed in response to LPS across multiple different species and tissues (Table S4). Eighty-four of the 145 LPS inducible miRNAs were found not be expressed above 1 tpm in BMEs. Of the 21 miRNAs that we identified as being differentially expressed in response to the Gram-positive *S. uberis*, only 9 of these (bta-let-7d, bta-let-7b, bta-mir-98, bta-miR-100, bta-mir-130a, bta-miR-193a, bta-miR-210, bta-miR-494, bta-miR-652) have also been reported to be differentially expressed in response to LPS in other species. Furthermore, 5 of these 9 (bta-miR-98, bta-miR-100, bta-miR-193a, bta-miR-210, bta-miR-494) show an inverse response to *S. uberis* infection in comparison to LPS. Most notably, bta-miR-100 and bta-miR-494, which were previously identified as up- and down-regulated, respectively, in mouse lung 6h post-stimulation with LPS, showed the inverse response at the same time-point in response to *S. uberis* infection [49], [50]. This would suggest that the miRNA response to Gram-positive bacteria may be markedly different to Gram-negative.

### Predicted Targets of Down-regulated miRNAs are Enriched for Genes with a Role in Innate Immunity

**Figure 7:**
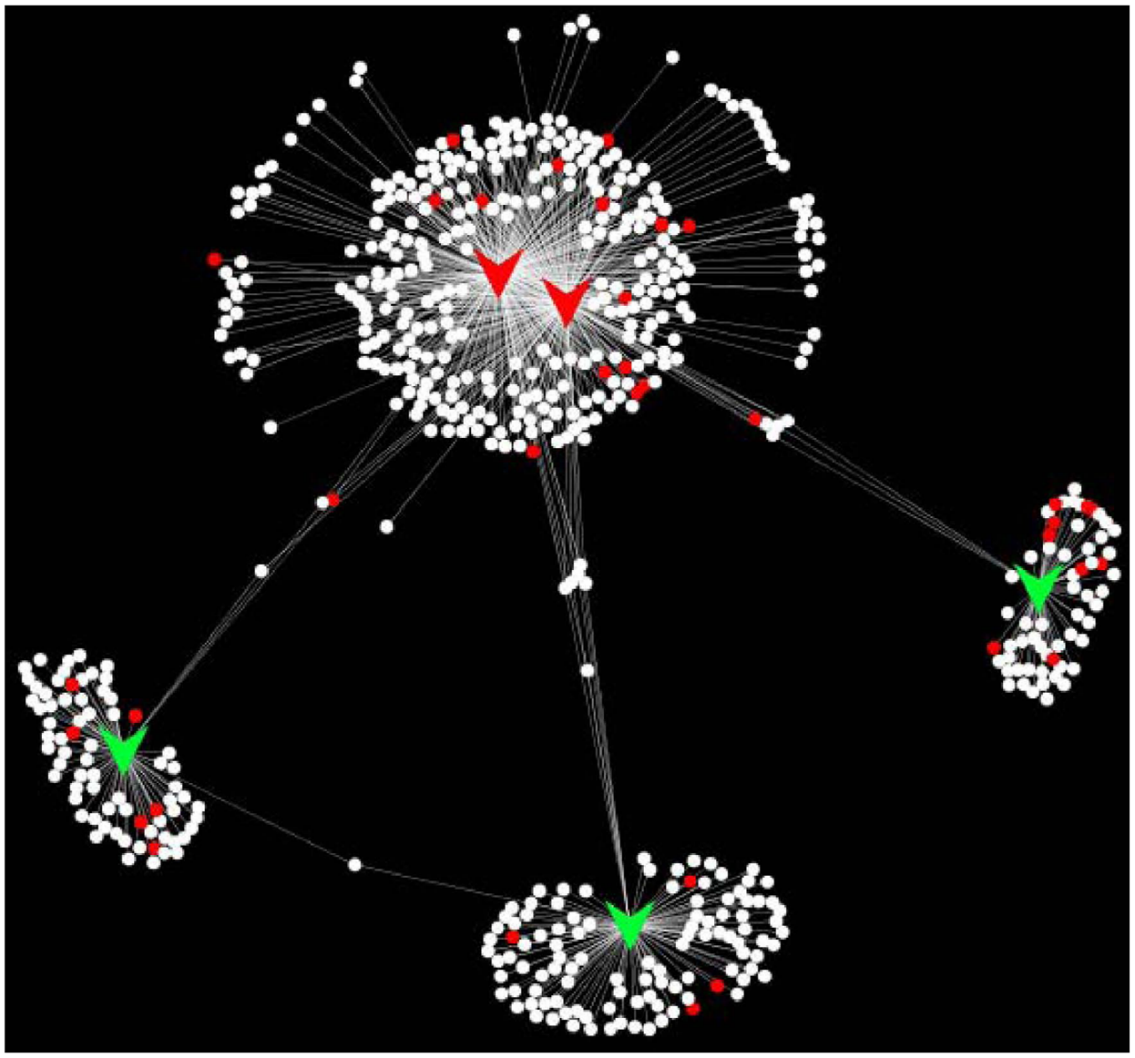
A network of miRNAs (arrow shapes) that were identified as being differentially expressed in BMEs at 4 hours post-infection with *S. uberis* and their predicted target genes (circles). Red arrow shapes represent up-regulated miRNAs at 4 hpi; green down-regulated. Red circles represent target genes that are annotated by www.innatedb.com as having a role in innate immunity. The network was constructed and visualised in Cytoscape v2.8.2. [69].

Target genes that are potentially regulated by differentially expressed miRNAs in response to *S. uberis* infection at 2, 4 and 6 hpi were predicted using two computational approaches, miRanda [33], [51] and TargetScan [34], [35], [36]. Given the high false positive rates for miRNA target prediction, we identified only those potential target genes that were predicted by both methods (Table 2). Target genes that were not corroborated by one of the two methods were discarded. In total 1,417 unique genes were predicted to be targeted by differentially expressed miRNAs (Table S5). This resulted in 2,491 miRNA-target interactions; 477 of these were targeted by down-regulated miRNAs; 1,921 were targeted by up-regulated miRNAs; and 93 were targeted by both up and down-regulated miRNAs. Because of the difficulties in accurately predicting miRNA targets, it is more appropriate to examine whether broad functional categories of genes are statistically over-represented among predicted target genes, rather than focusing on individual gene predictions. Statistical analysis (Hypergeometric test) revealed that the predicted target genes of down-regulated miRNAs at 4 and 6 hpi were significantly enriched (P = 0.01) in genes annotated by www.innatedb.com [38] as having a role in innate immunity (Figure 7). The predicted target genes of up-regulated miRNAs were not enriched for a role in innate immunity suggesting that up and down-regulated miRNAs target different processes in response to *S. uberis* infection.

**Table 2:** miRNA target predictions by miRanda and TargetScan and their intersect.

Name	MiRanda Targets(default)	TargetScan = (PCT>0.9)	TargetScan = (Quantile>0.9)	Number ofIntersectingTargets
bta-let-7b	6377	576	58	311
bta-let-7d	5637	576	53	290
bta-let-7e	5447	576	60	280
bta-mir-98	5095	576	58	274
bta-mir-185	5696	0	338	177
bta-mir-494	2952	0	336	151
bta-mir-200c	3362	74	201	123
bta-mir-29c	5131	179	76	115
bta-mir-29b-2	5394	179	76	114
ENSBTAG00000047296	5523	0	204	108
bta-mir-29e	5488	0	174	93
bta-mir-708	6414	0	179	84
bta-mir-210	4012	0	206	83
bta-mir-193a	3337	0	160	76
bta-mir-130a	2630	87	76	63
bta-mir-24-2	2157	0	107	45
bta-mir-2342	4296	0	64	37
bta-mir-128-2	4266	83	0	33
bta-mir-128-1	4266	83	0	33
bta-mir-100	946	0	13	1
bta-mir-652	0	0	0	0

Pathway analysis of the predicted gene targets of up-regulated miRNAs (at 4 and 6 hpi), revealed that pathways which have been previously implicated in mastitis are statistically enriched among the predicted gene targets of up-regulated miRNAs (Table 3). These pathways include MAPK signalling; Cytokine-Cytokine Receptor Signalling and the JAK-STAT Signalling Pathway. The MAPK signalling pathway, for instance, has been identified as one of the top canonical pathways highlighted in a microarray study examining the bovine mammary tissue response to mastitis, 20 hpi with *S. uberis* [52]. Many of the other pathways that we identified as statistically enriched among the predicted gene targets of up-regulated miRNAs were also highlighted as significant in this previous microarray study.

**Table 3:** Pathway analysis of the predicted target genes of up-regulated miRNAs 4 and 6 hours post-infection.

KEGG Pathway	FDR 4 hpi	FDR 6 hpi
MAPK signalling pathway	4.15E-33	2.79E−21
Cytokine-cytokine receptor interaction	4.71E-08	1.96E−32
Axon guidance	ns	3.35E−11
Calcium signalling pathway	ns	1.15E−06
MTOR signalling pathway	ns	1.36E−06
Colorectal cancer	ns	1.81E−06
Insulin signalling pathway	ns	3.69E−06
Jak-STAT signalling pathway	ns	3.56E−05
Fatty acid biosynthesis	ns	4.86E−05

* FDR = false discovery rate.

* hpi = hours post-infection.

Taken together, these analyses strongly suggest that miRNAs that are differentially expressed during infection of BMEs with *S. uberis* are key regulators of the host response to this pathogen.

### MicroRNA isomiRs

MicroRNA isomiRs are heterogeneous variants of canonical miRNA species, which are, increasingly, being suggested to be of functional importance [53]. It has been suggested that these miRNA variants can be cell type specific, have functional differences, and vary in their response to biological stimuli [54]. Evidence suggests that although isomiRs show similar expression patterns to their equivalent canonical miRNA, their targets can vary [55]. Deletions at both the 5′ and 3′ end of isomiRs may change the specificity of the seed binding region effecting miRNA function.

We have found that the expression of isomiRs was common for the majority of BME expressed miRNAs (Table 4). 100 known miRNAs were found to have at least one isomiR expressed at a level of >100 reads and more than 1,000 different isomiRs were identified. Notably, in 40% of cases at least one isomiR was more highly expressed than the miRbase consensus sequence, suggesting that the isomiR should in fact be annotated as the consensus. On further examination, we found that isomiR ‘nibbling’ was 1.4 more times likely than post-transcriptional additions and isomiR editing was 2.3 times more likely to be 3′ modified than 5′ modified, agreeing with current literature [54]. That said, almost 40% of isomiRs were 5′ modified, potentially impacting on which targets they regulate, though the majority of 5′ modified isomiRs were expressed at low levels. Further work is required to determine whether isomiRs have a functional role in response to infection.

**Table 4:** Analysis of isomiR heterogeneity across 24 miRNAseq samples.

Sample	# miRs withisomiRs*	# isomiRs	# longer than consensus	# shorter than consensus	# 5′ modified	# 3′ modified	# cases where isomiR expressed morehighly than consensus
1 hour control (replicate 1)	78	592	185	276	213	494	30
1 hour control (replicate 2)	108	1159	354	520	437	948	42
1 hour control (replicate 3)	95	818	242	372	260	694	36
1 hour infected (replicate 1)	103	1148	355	514	442	936	39
1 hour infected (replicate 2)	94	810	233	378	269	682	34
1 hour infected (replicate 3)	52	341	111	167	97	309	21
2 hour control (replicate 1)	104	1114	341	515	426	922	41
2 hour control (replicate 2)	94	999	307	460	390	828	40
2 hour control (replicate 3)	92	876	256	416	280	753	37
2 hour infected (replicate 1)	98	1075	321	513	437	881	37
2 hour infected (replicate 2)	91	870	259	418	346	713	40
2 hour infected (replicate 3)	91	829	249	366	297	676	37
1 hour infected (replicate 1)	114	1165	362	526	478	945	41
1 hour infected (replicate 2)	101	1086	310	482	411	879	39
1 hour infected (replicate 3)	141	1629	572	651	549	1341	59
2 hour control (replicate 1)	98	1048	314	446	343	867	37
2 hour control (replicate 2)	100	1008	313	467	410	824	32
2 hour control (replicate 3)	91	899	269	406	338	738	43
2 hour infected (replicate 1)	96	1056	321	495	419	881	40
2 hour infected (replicate 2)	97	1002	307	459	349	851	39
2 hour infected (replicate 3)	102	1062	330	504	441	881	38
4 hour control (replicate 1)	120	1245	448	533	397	1070	51
4 hour control (replicate 2)	119	1165	408	510	371	998	52
4 hour control (replicate 3)	137	1796	633	723	597	1500	63

* Only isomiRs present at >100 reads are shown.

### Novel miRNA Discovery

In addition to profiling the expression of known miRNAs, miRNAseq data can also be used to identify the expression of potentially novel miRNAs. To do this, miRNAseq data from this study was analysed using the software package, miRDeep2 [32]. We identified 21 high-confidence, putatively novel, bovine miRNAs that were independently predicted in multiple BME miRNAseq datasets (Table 5). Homology searching of the miRBase database (v 19) [3] using BLAST [56] identified that 2 of the novel miRNAs had 100% identity to known miRNAs in other species, ssc-miR-664-3p (pig) and hsa-miR-219-1 (human). Additionally 5 of the novel bovine miRNAs had significant homology with the bta-mir-2285 family. The bta-mir-2285 family has over 40 members spanning the entire bovine genome, [20]. Two additional novel miRNAs showed homology to the bta-mir-2284 family. The remaining miRNAs did not show significant homology to other known miRNAs in other species. However, given the very high read counts observed for several of these predicted miRNAs, and the fact that were independently predicted in multiple different samples, it would suggest that many of these predictions represent true novel bovine miRNAs.

**Table 5:** Putative novel bovine miRNAs discovered through miRDeep2 analysis of miRNAseq data from 24 bovine primary mammary epithelial cell samples.

Name *	Mature Sequence Best miRBase BLAST Hit(e-value <1)	# Samples miRNAis Predicted in	Mature Tag Count **	Predicted Mature Sequence
bta-mir-6537	N/A	7	272,924	gugggacgcgugcguuuu
bta-mir-6538	N/A	22	22,094	auagccaguuggggaagaaugc
bta-mir-6539	N/A	20	9,687	acgcaauucuucaaaaucuuagc
bta-mir-6540	N/A	16	2,840	aaaaacuggcagcuucauguaa
bta-mir-2285i-1	bta-miR-2285i	13	2,241	aaaacuggaacgaacuuuugggc
bta-mir-2285f-3	bta-miR-2285f	18	2,202	aaaaccugaaugaacuucuugg
bta-mir-2284z-8	bta-miR-2284z	15	2,013	uaaaaguuugguuggguuuuu
bta-mir-664b	ssc-miR-664-3p	20	1,736	uauucauuuaucucccagccuac
bta-mir-6541	N/A	8	1,501	uggagcggcugcacagagcgu
bta-mir-2285c-1	bta-miR-2285c	14	694	aaaaccugaagagacuuuuugg
bta-mir-6542	N/A	13	693	ugcuaccuagucugagugaguga
bta-mir-6544	N/A	18	332	uggugcucccuggagcugagc
bta-mir-6516	gga-miR-6516-5p	7	242	uuugcaguaacaggugugaac
bta-mir-219-1	hsa-miR-219-1-3p	3	173	agaguugagucuggacgucccg
bta-mir-6545	N/A	3	121	auggacugucaccugaggagc
bta-mir-2285m-6	bta-miR-2285m	2	107	aaaacccaaaugaacuuuuugg
bta-mir-2284b-1	bta-miR-2284b	5	86	aaauguucgcuuggcuuuuucc
bta-mir-2285f-4	bta-miR-2285f	2	64	agaaaguucauuuagguuuuuc
bta-mir-6546	N/A	4	52	cuuccucuuccgguuggcaga
bta-mir-6547	N/A	3	29	auucuccauuggauauaauagu
bta-mir-6643	gga-miR-6643-5p	2	21	cagggagggcaggggaggg

* Bovine miRNA names for the novel miRNAs have been assigned according to miRBase nomenclature rules.

** The number of reads aligning the mature miRNA.

## Discussion

In this study, we have used a next generation sequencing approach to profile the expression of bovine miRNAs at multiple time-points in primary bovine mammary epithelial cells (BMEs) infected *in vitro* with *S. uberis*, a causative agent of bovine mastitis. In comparison to previous NGS studies investigating the host miRNA response to infection, we have sequenced un-pooled miRNA libraries to a previously unprecedented sequencing depth from multiple replicates and controls across multiple time-points, allowing us to explore statistically significant temporal changes in miRNA expression in response to infection. Analysing over 450 million sequencing reads, we found that approximately 20% of known bovine miRNAs are expressed in BMEs. A similar diversity of miRNA expression has also been recently reported in other tissues, including bovine retinal microvascular endothelial cells (RMECs) [20] and in testicular and ovarian tissues [18]. As has also been reported in other studies, there is a significant dynamic range in the expression of known miRNAs in BMEs. A few miRNAs are expressed at very high levels, with the majority being expressed at low levels. The top 10 most highly expressed miRNAs account for >80% of all aligned reads and are highly conserved across species. Whether or not the other more lowly expressed miRNAs play a significant biological role remains an open question.

We have also found that the expression of isomiRs was common for many of the BME expressed miRNAs. Most significantly, in 40% of cases, at least one isomiR was more highly expressed than the miRbase consensus sequence, indicating that studies such as ours can also be used to improve miRNA annotation. In particular, changes to the 5′ of the consensus sequence could lead to dramatically different target genes being computationally predicted. We identified a large number of 5′ isomiRs although they were mostly expressed at low levels.

In addition to profiling known miRNAs, we have also analysed the sequencing data to identify potentially novel bovine miRNAs. Twenty-one high-confidence, putatively novel, bovine miRNAs were identified independently across multiple different samples. The mature sequences of two of the novel miRNAs were 100% identical to known miRNAs in other species. Seven of the other predicted miRNAs exhibited significant homology to two bovine miRNA families, bta-mir-2284 and bta-mir-2285.

Few studies have previously investigated temporal changes in global miRNA expression using an NGS approach [57], although several have used microarray technology [58], [59], [60], [61]. Thus far, many immune-relevant miRNAs have been identified as part of the host response to LPS stimulation [47], [48]. We completed a literature survey and identified more than 145 miRNAs across multiple different species and tissues that have been shown to be LPS responsive. In our study, the miRNA response to the Gram-positive *S. uberis* was markedly different to the reported LPS miRNA response. Over the 6 hour time-course, we identified 21 known bovine miRNAs as significantly differentially expressed in response to the *S. uberis* challenge. Only 9 of these miRNAs have also been reported to be differentially expressed in response to LPS. For those in common, an inverse pattern of expression was observed in 5 of the 9 cases suggesting that the miRNA response to Gram-positive bacteria may be markedly different to Gram-negative. Further global studies of the miRNA response to Gram-positive and Gram-negative bacteria in the same tissue at the same time-points will be required to confirm this.

It is notable that we found most miRNAs were differentially expressed at different time-points post-infection, suggesting that miRNAs exhibit rapid dynamics in their temporal expression. It is also notable that the majority of miRNAs that we report as being differentially expressed exhibit relatively subtle changes in gene expression in response to infection. This subtle change in expression is in line with existing literature and strengthens the hypothesis that miRNAs are fine-tuners of gene expression [48], [62]. For example, miR-let-7d, miR-652 and miR-494 demonstrated similar levels of differential expression 6 hours post LTA stimulation in mouse tissues [50].

Computational analysis revealed that the predicted target genes of *S. uberis* down-regulated miRNAs were statistically enriched for roles in innate immunity. This would suggest that these miRNAs may significantly regulate the sentinel capacity of mammary epithelial cells to mobilise the innate immune system [63]. Pathway analysis of the predicted targets of up-regulated miRNAs has also identified the statistical over-representation of several pathways previously implicated in the host response to mastitis, such as the MAPK, JAK-STAT and other cytokine signaling pathways. Furthermore, several of the differentially expressed miRNAs have been shown to have roles in the immune systems of other species. For example, bta-let-7 miRNAs were up-regulated at both 4 and 6 hours post-infection with *S. uberis*. The let-7 family has been extensively described in the literature for having a role in immunity. The down-regulation of let-7 family members, for example, was shown to promote expression of IL-10 and IL-6 in HeLa cells infected with *Salmonella enterica* serovar Typhimurium [64]. The observed up-regulation of let-7 miRNAs in our study may lead to the repression of anti-inflammatory cytokines to promote innate immunity.

We also report the down-regulation of two other miRNAs, bta-miR-29b-2 and bta-miR-130a, both of which have known roles in immunity and infection in other species. miR-29a/miR-29b down-regulation has been demonstrated to facilitate IFN-γ up-regulation in NK cells and T_H_1 cells [65], [66]. IFN- γ is well known as an innate inflammatory mediator and its secretion promotes host resistance against viral and intracellular bacteria. Furthermore, IFN-γ mRNA expression has been demonstrated in human mammary epithelial cells [67], suggesting that this may be a relevant target in our model. LPS induced TNF-α expression in neonatal and adult monocytes has been shown to be greatly suppressed by the induction of miR-130a [68]. Taken together, the evidence strongly suggests that the differentially expressed miRNAs identified in this study are likely central regulators of the innate immune response to *S. uberis* and thus represent potential therapeutic targets or novel biomarkers of infection and inflammation.

## Supporting Information

Figure S1 **Read coverage along chromosome 26 (25 nt windows).** The higher the read density the darker the red colour. Green regions represent positions where the read density is < 5 reads, Grey = no reads.
(TIF)

Figure S2 **Fold changes of differentially expressed miRNAs at 4 hours post-infection (hpi).**
(TIF)

Figure S3 **Fold changes of differentially expressed miRNAs at 6 hours post-infection (hpi).**
(TIF)

Table S1 **Summary read statistics, barcodes and adaptor sequences for each mammary epithelial cell miRNAseq library.**
(XLS)

Table S2 **RNA integrity, quantity, 28S/18S ratio, and miRNA quantity for each of the 24 samples.**
(XLS)

Table S3 **miRNAs that are expressed at ≥1 tag per million in bovine mammary epithelial cells.**
(XLS)

Table S4 **Summary of lipopolysaccharide (LPS) responsive microRNAs.** 145 miRNAs that have been identified as being differentially expressed in response to LPS across multiple different species and tissues.
(XLSX)

Table S5 **Genes predicted to be targeted by differentially expressed miRNAs.**
(XLS)
