# Supplementary figures and images for: Next Generation Sequencing Reveals the Expression of a Unique miRNA Profile in Response to a Gram-Positive Bacterial Infection
Source: PLoS One. 2013 Mar 5;8(3):e57543. doi: 10.1371/journal.pone.0057543 (PMC3589390; doi:10.1371/journal.pone.0057543)

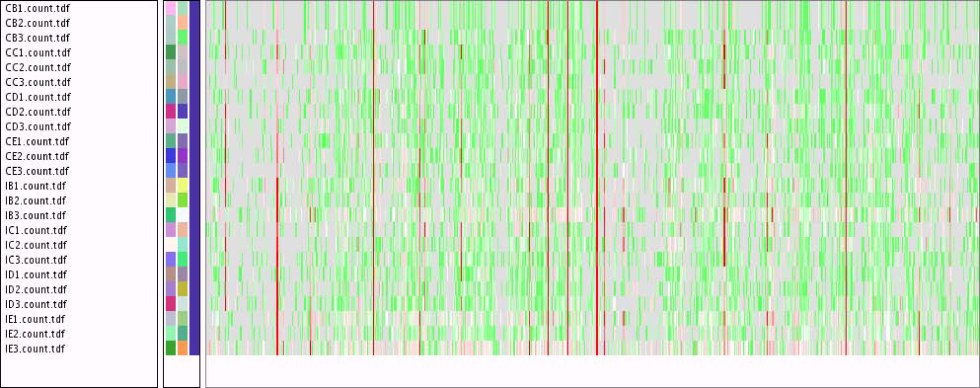

Supplement: Figure S1 — Read coverage along chromosome 26 (25 nt windows). The higher the read density the darker the red colour. Green regions represent positions where the read density is < 5 reads, Grey = no reads. (TIF) [file pone.0057543.s001.tif]

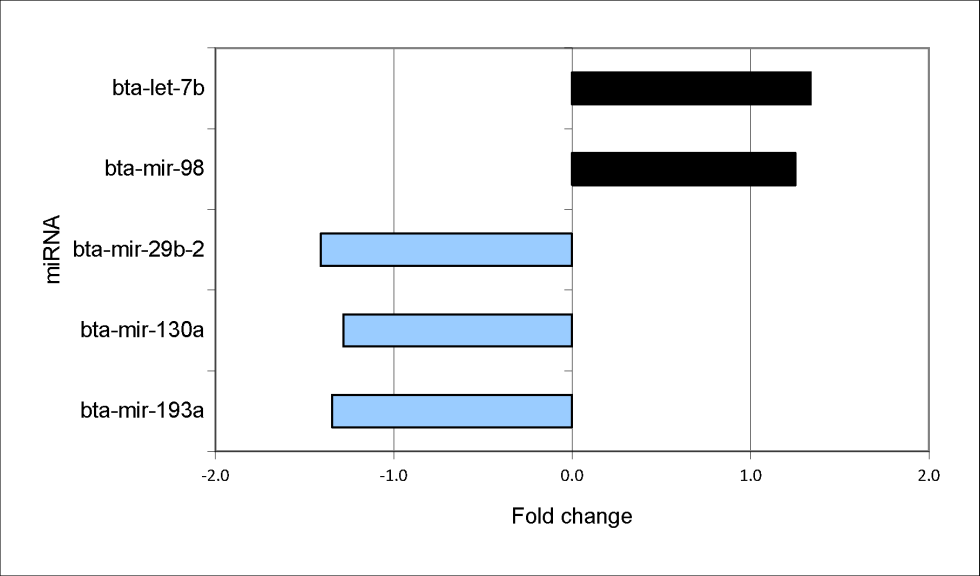

Supplement: Figure S2 — Fold changes of differentially expressed miRNAs at 4 hours post-infection (hpi). (TIF) [file pone.0057543.s002.tif]

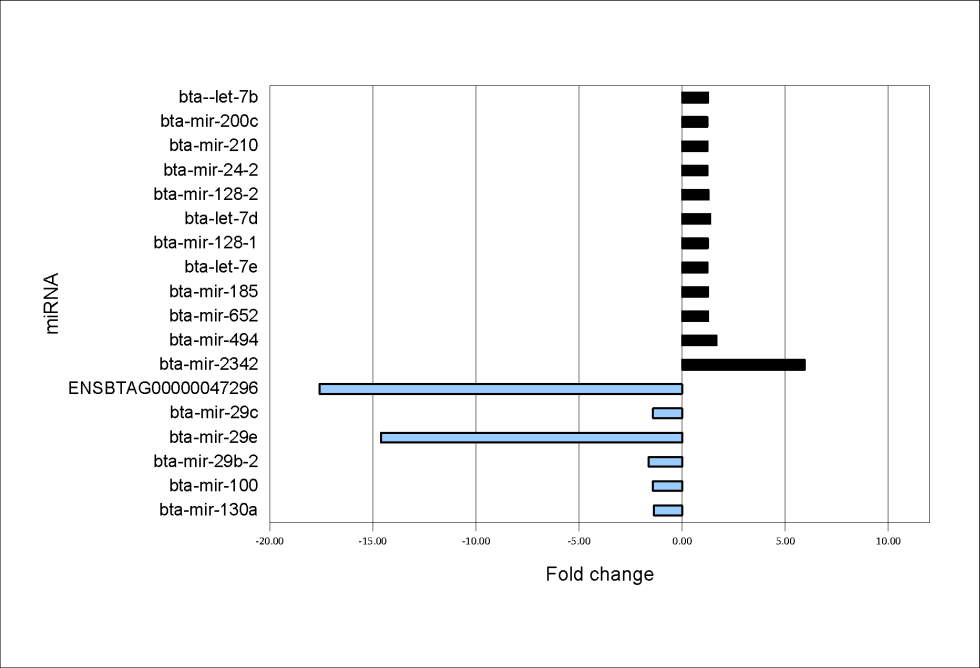

Supplement: Figure S3 — Fold changes of differentially expressed miRNAs at 6 hours post-infection (hpi). (TIF) [file pone.0057543.s003.tif]
